# High intimal flap mobility assessed by intravascular ultrasound is associated with better short-term results after TEVAR in chronic aortic dissection

**DOI:** 10.1038/s41598-019-43856-6

**Published:** 2019-05-13

**Authors:** Julia Lortz, Maria Papathanasiou, Christos Rammos, Martin Steinmetz, Alexander Lind, Konstantinos Tsagakis, Thomas Schlosser, Heinz Jakob, Tienush Rassaf, Rolf Alexander Jánosi

**Affiliations:** 1 Department of Cardiology and Vascular Medicine, West- German Heart and Vascular Center Essen, University of Duisburg-Essen, Essen, Germany; 2 Department of Thoracic and Cardiovascular Surgery, West- German Heart and Vascular Center Essen, University of Duisburg-Essen, Essen, Germany; 3 Department of Diagnostic and Interventional Radiology and Neuroradiology, University Hospital Essen, University of Duisburg-Essen, Essen, Germany

**Keywords:** Cardiology, Interventional cardiology

## Abstract

Thoracic endovascular aortic repair (TEVAR) in chronic aortic dissection remains controversial. We analysed whether a high intimal flap mobility (IFM) of the dissection membrane has an impact on aortic remodelling after TEVAR in chronic Type B aortic dissection. Patients undergoing TEVAR with intravascular ultrasound (IVUS) were analysed and IFM was calculated. High IFM was defined as maximum flap amplitude >3 mm. For determining aortic remodelling, the degree of true lumen (TL) expansion was analysed in the last available follow-up CT. Fifty-two patients (63.6 ± 15.4 years) with a mean follow-up of 26.6 ± 20.7 months were analysed. The mobile flap group (n = 29) showed higher absolute TL expansion at the distal stent-graft (5.9 ± 3.1 vs. 3.3 ± 5.4 mm; *p* = 0.036) and a higher increase in TL diameter (18 ± 10 vs. 9 ± 15%; *p* = 0.017) compared to the non-mobile group (n = 23). Basic TEVAR-related outcome characteristics were comparable, but the mobile intimal flap group showed a lower re-intervention rate (3 vs. 8pts.; *p* = 0.032) in chronic dissections. High IFM in chronic Type B aortic dissection is linked to improved aortic remodelling and is associated with a lower re-intervention rate over time. IVUS assessment of IFM in chronic Type B aortic dissection might be helpful in identifying patients with better remodelling after TEVAR.

## Introduction

The treatment of chronic Type B aortic dissection is challenging due to the heterogeneous nature of the morphological disease pattern. Thoracic endovascular aortic repair (TEVAR) is a potential treatment option^1,2^, but which subgroups of patients will benefit most from this approach remains the subject of investigations^3,4^. The different stages in chronic Type B aortic dissection might be one potential explanation for this heterogeneous disease since definitions of chronic dissection vary widely^5–7^. One potential approach to address the different stages of disease grade might be the assessment of intimal flap mobility. The progress of aortic disease is attended by changes in intimal flap including loss of mobility, tending to a straighter and thicker flap^8^. Preserved mobility of the intimal flap underlies changing flow conditions during the cardiac cycle as the aorta is a dynamic structure that oscillates^9^. The diagnostic information regarding the intimal flap dynamics might be of interest for the interventionalist to narrow down the therapeutic window for TEVAR or to identify patients at risk with impaired outcome due to the low remodelling capacity of the aorta.

Several methods are available to assess intimal flap mobility. Computer tomography (CT) is associated with radiation and the intimal flap assessment needs to be planned in advance^10^. Intravascular ultrasound (IVUS) allows real-time assessment^11^ and provides information regarding dynamics of the intimal flap mobility within the cardiac cycle. However, the use of IVUS is limited due to cost inefficiency and the relative lack of availability.

Studies on evaluation of intimal flap dynamics via IVUS are scarce and the potential benefits of IVUS assessment are unclear. We evaluated the feasibility of intimal flap mobility assessed by IVUS in patients with chronic Type B aortic dissection. We compared true luminal expansion as a marker for aortic remodelling, and outcomes at follow-up between patients with mobile and non-mobile intimal flaps.

## Methods

### Study design and patient characteristics

In this single-centre retrospective study, we evaluated the impact of intimal flap mobility assessed by IVUS on the outcomes of patients with chronic Type B aortic dissection undergoing TEVAR. All patients with chronic Type B aortic dissection who underwent TEVAR with IVUS from August 2008 to November 2016 at our institution were examined.

The indications for treatment included persistent pain, refractory hypertension, acute limb, intestinal, or renal ischemia, and a maximum descending or thoracic aortic diameter of 50 mm. Those with chronic Type B aortic dissection and both IVUS and CT before TEVAR were identified and clinical data including IVUS and CT measurements were obtained. Aortic dissection was considered to be chronic after 14 days from symptom onset^5^.

Patients were divided into two groups depending on the intimal flap mobility assessed by IVUS. All patients were treated at a major tertiary referral centre by a team of interventional cardiologists, cardiothoracic surgeons, and anaesthesiologists with significant expertise in acute aortic syndromes.

The study was approved by the local ethics committee of the University of Duisburg-Essen and was conducted in accordance with the principles of the Declaration of Helsinki. Patient records were de-identified and analysed anonymously. The local ethics committee approved the retrospective analysis of patient data without the need to obtain patient consent.

### IVUS procedure and TEVAR follow-up

IVUS studies were performed at the time of TEVAR using the Visions PV 0.035 catheter system (Volcano, San Diego, CA, USA). The IVUS catheter uses 10-MHz frequency ultrasound and has a maximum imaging diameter of 60 mm. A pigtail catheter was inserted into the ascending aorta. Over a long guide wire introduced over the catheter, the IVUS catheter was positioned in the ascending aorta close to the aortic valve. The IVUS probe was manually retracted to a point corresponding to the distal part of the sheath. Grayscale images were simultaneously acquired digitally. Gyrating movements were used in an attempt to gain an optimal cross-sectional aortic image. The cardiologist performing these IVUS scans had previous experience acquiring IVUS measurements from more than 50 aortas. The timing of definite TEVAR was dependent on the underlying aortic disease, based on which, it was classified as elective or emergency TEVAR procedures. The following stent grafts were used: Valiant and Valiant-Captivia (Medtronic, Minneapolis, MN, USA), Relay (Bolton Medical, Barcelona, Spain), and GORE TAG (W.L. Gore & Ass., Flagstaff). The chosen stent graft size either included 10% of oversizing (Medtronic, Bolton Medical) or was decided based on the range specified by the manufacturer’s guidelines (GORE).

The TEVAR procedure was performed in the cardiac catheterization laboratory by a team of interventional cardiologists, cardiothoracic surgeons, and anaesthesiologists under general anaesthesia with mechanical ventilation. The procedure was performed under sterile conditions and intravenous antibiotics (ceftriaxone) were administered prior to its initiation. After surgical exposure of the femoral artery, a standard 6-French arterial sheath was administered and 5000 IU of heparin was injected. An angiogram was obtained using a graduated 6-French pigtail catheter with metallic markers to determine the anatomical conditions, including location of side branches (i.e. LSA) and to establish the strategy for optimal stent-graft landing zones. Additionally, a 6-French pigtail catheter was advanced from the left radial artery to enable intra-procedural angiography. The stent-graft delivery system was advanced over an ultra stiff 0.035 in guide wire (Meier Back-Up, Boston Scientific, Oakland, CA, USA). The stent-grafts were positioned in the thoracic aorta under fluoroscopic guidance. Before the stent-graft was deployed, systolic blood pressure was lowered to 50 mmHg using intravenous sodium nitroprusside to prevent inadvertent downstream displacement of the stent-graft during delivery. Immediate procedural success was evaluated via angiography. No additional heparin or antiplatelet medications were administered following completion of the procedure.

### CT study

Contrast-enhanced, electrocardiogram-triggered, high-resolution (≤1.5-mm slice thickness) CT angiography (CTA) was performed either on a 64-row multidetector scanner at our institution (Somatom Definition; Siemens Healthcare, Forchheim, Germany) or was previously performed at the referring outside facility. The following examination protocol is used by our institution. Continuous scans covering the entire aorta, including the proximal supraaortic vessels down to the groin were obtained. Iodinated contrast (120 to 140 mL) was continuously injected into the right antecubital vein via an 18-G catheter at an infusion rate of 3.5 mL/s. To ensure maximum contrast concentration in the aorta, a circular region of interest (ROI) was placed in the ascending aorta. As soon as the signal intensity in the ROI reached a threshold of 120 Hounsfield units, the patient was instructed to maintain an inspiration breath hold, at which point data acquisition commenced. A second late arterial phase scan was performed after a delay of 15 s, covering the same area. Imaging was performed prior to TEVAR, the day after TEVAR, and at a later unspecified (non-standardized interval) follow-up time.

### Intimal flap characterization

**Figure 1:**
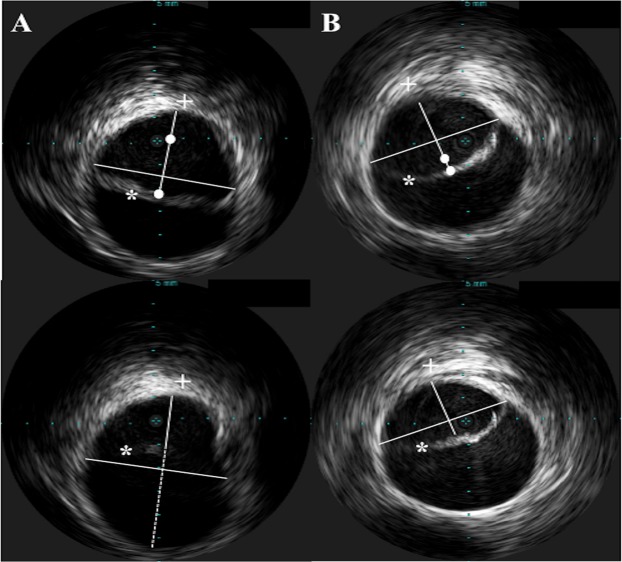
Example of mobile (**A**) and non-mobile (**B**) intimal flaps in chronic Type B aortic dissection. Amplitude of intimal flap mobility (white dot) was assessed by connecting the endpoints of the dissected flap and drawing a perpendicular line through the mid point of this line. The minimum and maximum diameter between the intimal flap (*) and the free aortic wall (+) defined the maximum amplitude of intimal flap mobility.

To assess intimal flap mobility, we analysed IVUS imaging before TEVAR. The intimal flap mobility was considered high with an amplitude of >3 mm (mobile intimal flap), and low with an amplitude of ≤3 mm (non-mobile intimal flap). We classified non-mobility as ≤3 mm and a real-time intimal flap movement of >3 mm. The cut-off value for mobility visualized by IVUS was defined as <10% of the total aortic diameter, which is more comparable to pulsatility. Three consecutive measurements were made at the level with the highest mobility by two independent observers and the mean value was recorded. Observers were blinded to the patient and study details. If more areas with high mobility were detected, each area was assessed separately as described above and the one with the highest mobility was selected for analysis. We measured the maximum and minimum diameter of the true lumen short axis by connecting the endpoints of the dissected flap and drawing a perpendicular line through the mid-point of this line (Fig. 1).

The following calculations were used to describe intimal flap characterization:

- Max. diameter free aortic wall (FAW) – intimal flap (IF): Maximum diameter between FAW and IF
- Min. diameter FAW – IF: Minimum diameter between FAW and IF
- Max. amplitude of IF change: (Max. diameter FAW – IF) – (Min diameter FAW – IF)
- Relative true lumen (TL) max. decrease: (Min. diameter FAW – IF)/total aortic diameter
- Δ TL shift: (Max. amplitude of IF change)/total aortic diameter.

### True lumen expansion

The CT examinations were performed before TEVAR, the day after TEVAR and at last available follow-up. To assess the distal stent end, the CT procedure that was performed the day after TEVAR served as a benchmark for locating the level of the inferior edge of the stent graft.

An additional cross-sectional analysis was performed at the level of the pulmonary artery and the diaphragm in both CT scans. The minimum and maximum diameters of the true lumen were assessed in the pre-treatment and follow-up CT scans, and the mean diameters were calculated as previously described^12^. For follow-up values, we evaluated the most recently acquired CT scans. All measurements were made by two independent observers in a blinded manner.

### Statistical analysis

Continuous variables are presented as means ± standard deviation, while categorical variables are presented as frequencies and percentages. The Mann-Whitney U test and chi-square test were used for the comparison of categorical variables. Student’s *t*-test was used for the analysis of continuous variables. Values of p < 0.05 were considered statistically significant.

All data and statistical analyses were performed using SPSS 24 (Chicago, IL, USA) for Mac and Microsoft Excel 2011 for Mac.

## Results

### Study population and interventional characteristics

Fifty-two patients undergoing TEVAR and preliminary IVUS due to chronic Type B aortic dissection between August 2008 and November 2016 were analysed. Twenty-nine patients had a mobile intimal flap with a maximum amplitude of >3 mm assessed in the IVUS procedure previous to TEVAR, and 23 showed an impaired intimal flap mobility ≤3 mm. The relevant patient demographics are shown in Table 1. No relevant differences in comorbidities were found between the two groups. Basic interventional characteristics did not differ in both groups: The stent-graft size (mobile: 36.3 ± 7.8 mm, non-mobile: 34.3 ± 6.8 mm; *p* = 0.309) and length (mobile: 158.2 ± 52.3 mm, non-mobile: 169.2 ± 37.2 mm; *p* = 0.398) were comparable between the two groups. We also saw a similar distribution of stent-graft types in both groups: the most commonly implanted stent-graft were the Medtronic Valiant or Valiant-Captivia prosthesis (n = 14 vs. n = 10), followed by Bolton Relay (n = 13 vs. n = 9) and GORE TAG (n = 2 vs. n = 4; *p* = 0.5).

**Table 1:** Patient demographics with chronic Type B aortic dissection.

	Mobile flap	Non-mobile flap	*p* value
	n = 29	n = 23	
Age (years), mean ± SD	61.6 ± 17.2	66.2 ± 12.7	0.295
Men	7 (24)	10 (43)	0.140
Onset symptoms to intervention (days), mean ± SD	21.5 ± 6.5	26.0 ± 8.5	0.032*
Subacute dissections*	25 (86)	16 (70)	0.144
Previous aortic surgery	5 (17)	6 (26)	0.438
Coronary artery disease	15 (52)	9 (39)	0.366
Diabetes mellitus Type 2	4 (14)	2 (9)	0.567
Chronic kidney disease	5 (17)	5 (22)	0.683
Peripheral artery disease	8 (28)	3 (13)	0.202
Smoking	13 (45)	10 (43)	0.922
Hypercholesteremia	16 (55)	9 (39)	0.250

SD, standard deviation. Categorical data are presented as number (%). *Assessed 8 to 30 days after symptom onset according to the International Registry of Acute Aortic Dissections IRAD registry^7^.

### Intimal flap characteristics

Approximately half of the patients with non-mobile intimal flap had no measurable amplitude (n = 12). Four patients had amplitude of 1 mm, six patients with 2 mm and one with 3 mm. The amplitudes in the mobile flap group ranged from 5–12 mm with a mean of 7 mm.

While the maximal true lumen compression did not differ between both groups (Min diameter FAW – IF), the mobile flap group showed a significantly greater true lumen expansion during one cardiac cycle due to the high mobility of intimal flap (Table 2).

**Table 2:** Intimal flap characterization by IVUS before TEVAR.

	All	Mobile flap	Non-mobile flap	*p* value
	n = 52	n = 29	n = 23	
Max diameter FAW – IF (mm)	2.0 ± 0.8	2.2 ± 0.8	1.8 ± 0.8	0.049*
Min diameter FAW – IF (mm)	1.4 ± 0.6	1.5 ± 0.7	1.7 ± 0.8	0.432
Max amplitude of IF change (mm)	0.6 ± 0.2	0.7 ± 0.2	0.1 ± 0.1	0.001*
Relative TL max decrease (%)	56 ± 20	62 ± 18	49 ± 20	0.013*
Δ TL shift (%)	39 ± 27	65 ± 31	5 ± 4	0.001*
Total aortic diameter (mm)	36.0 ± 6.8	36.6 ± 6	35.6 ± 7.5	0.595

FAW, free aortic wall; IF, intimal flap; IVUS, intravascular ultrasound; TEVAR, thoracic endovascular aortic repair; TL, true lumen; Data are presented as mean ± standard deviation.

The mobility of the dissection membrane led to a greater true luminal loss and true lumen shift in the mobile flap group during every cardiac cycle.

### True lumen expansion at follow-up

The overall mean follow-up time was 26.6 ± 20.7 months (median 23 months) with a minimum of 2.7 months and a maximum of 79.9 months. The follow-up time between both groups did not differ (mobile flap: 27.1 ± 21.8 months, non-mobile flap 25.9 ± 19.7 months; *p* = 0.837).

The assessed true lumen expansion at follow-up is shown in Table 3. Mean true lumen diameter assessed in the follow-up CT was significantly higher in patients with mobile flap (mobile: 29.8 ± 6.3 mm, non-mobile: 26.4 ± 4.9 mm; *p* = 0.039) with comparable aortic diameters of the distal stent end at follow-up (mobile: 37.7 ± 8.3 mm, non-mobile: 38.3 ± 6.8 mm; *p* = 0.808). Comparison of the total aortic diameters 1 cm distal to the distal stent end revealed no significant differences in the absolute aortic diameter (mobile: 37.1 ± 8.6 mm, non-mobile: 39.4 ± 7.0 mm; *p* = 0.322). However, a significant increase in total aortic diameter was observed compared to the original value in the non-mobile group (mobile: −0.7 ± 0.7 mm, non-mobile: 1.1 ± 0.9 mm; *p* = 0.001).

**Table 3:** Increase/expansion of true lumen (TL) from pre TEVAR to follow-up at different aortic levels assessed by CT.

	Mobile flap	Non-mobile flap	*p* value
	n = 29	n = 23	
TL expansion absolute, DSE (mm)	5.9 ± 3.1	3.3 ± 5.4	0.036
TL expansion absolute, PA (mm)	5.8 ± 2.7	3.7 ± 5.1	0.052
TL expansion absolute, DP (mm)	5.9 ± 2.9	3.2 ± 5.5	0.028
Increase in TL diameter to AD, DSE (%)	18 ± 10	9 ± 15	0.017
Increase in TL diameter to AD, PA (%)	17 ± 9	10 ± 14	0.024
Increase in TL diameter to AD, DP (%)	18 ± 9	9 ± 15	0.013

AD, aortic diameter; CT, computed tomography; DP, diaphragm; DSE, distal stent end; PA, pulmonary artery; TEVAR, thoracic endovascular aortic repair; TL, true lumen. Data are presented as mean ± standard deviation.

### Patient outcome

The assessment of patient outcomes at follow-up included the most important TEVAR-associated characteristics including type I endoleak and re-intervention rate. No differences in type I endoleak were detected. The overall re-intervention rate was 21.2%, which turned out to be higher in the non-mobile flap group (Table 4). The time to re-intervention tended to be longer in the mobile flap groups, without reaching statistical significance. The overall mortality was low (9.6%) and did not differ between both groups. Reasons for death were gastrointestinal bleeding (n = 0 vs. n = 1), non-procedure-associated stroke (n = 0 vs. n = 1), septic shock (n = 1 vs. n = 1) and cancer-related disease (n = 0 vs. n = 1).

**Table 4:** Related outcome characteristics after TEVAR at follow-up.

	Mobile flap	Non-mobile flap	*p* value
	n = 29	n = 23	
Type I endoleak	2 (7)	1 (4)	0.695
Total remodeling	8 (28)	1 (4)	0.028
Re-intervention	3 (10)	8 (35)	0.032
Time to re-intervention (months)	26.7 ± 22.6	13.6 ± 10.3	0.198
Mortality	1 (3)	4 (17)	0.090

TEVAR, thoracic endovascular aortic repair. Data are presented as mean ± standard deviation or n/%.

Reasons for re-intervention were type I endoleak (n = 2 vs. n = 1), distal stent-graft induced new entry (n = 1 vs. n = 1), retrograde dissection (n = 0 vs. n = 1), stent-graft migration (n = 0 vs. n = 1), malperfusion and need for re-intervention (n = 0 vs. n = 4). All re-interventions were discussed between the aortic team members (which included a interventional cardiologist, surgeon and anesthesiologist) and decisions for re-intervention were made jointly. All re-interventions included distal (n = 7) or proximal (n = 4) stent-graft extensions.

The number of cases of total remodeling was significantly higher in the mobile flap group (28% vs. 4%).

## Discussion

We demonstrated that IVUS assessment of a highly mobile dissection membrane prior to TEVAR is associated with improved aortic remodelling and a lower re-intervention rate in patients with chronic Type B aortic dissection. Interventional treatment in these patients might be beneficial, whereas patients with low intimal flap mobility may need intensified follow-up.

The debate regarding the interventional approach in the treatment of chronic Type B aortic dissection is still controversial. The simple sealing of the proximal entry tear for rerouting the falsely flowing blood into the true lumen alone does not ensure a satisfactory outcome. One potential reason might be the impaired remodelling capacity of the aortic tissue compared to earlier stages due thickening of the aortic wall and intimal flap over time^1,13^. This is in line with our finding that the time between symptom onset and intervention was longer in patients with a non-mobile flap. According to the guideline definition for chronic aortic dissections that was used in the present study^5^, all patients were classified as in the chronic phase. The International Registry of Acute Aortic Dissections (IRAD) differentiates an additional subacute phase (8–30 days between symptom onset and intervention), in which higher survival and better aortic remodeling was observed compared with the chronic phase^7^. This may indicate an early-onset conversion process of the dissection flap. Nevertheless, in the present study, the time between symptom onset and intervention was significant longer in the non-mobile group. The absolute number of subacute patients according to IRAD was comparable between groups.

IVUS is a well-established method for the diagnosis of coronary dissections^14,15^ and the peripheral vasculature^16^, but studies addressing aortic diseases are sparse. Intimal flap mobility assessment by IVUS has not been described yet. Intimal flap mobility of the carotid artery has been assessed by IVUS in two patients undergoing carotid artery angioplasty^17^. A major advantage of IVUS is that it combines the detection of underlying aortic disease with precise localization of pathologies^18,19^ and dynamic changes in the aortic tissue in real-time^20^. Other tools for intimal flap assessment include transesophageal ultrasound and magnetic resonance imaging. The use of transesophageal ultrasound would additionally need invasive access and is barely applicable for complete assessment of the thoracic aorta. Magnetic resonance imaging offers dynamic quantification of blood volume and velocity during the cardiac cycle in chronic Type B aortic dissection^21^, but might be unreliable in patients with complex flow patterns or hemodynamic instability.

CT is the state-of-the-art technique for the diagnosis of aortic diseases. Planning of the scan protocol, including ECG-gating is required, which is always related to a higher radiation dose. Previous CT findings have described the association of intimal flap mobility with a comparable decrease in the true lumen up to 29% during the cardiac cycle and are in line with our findings^9^.

The increasing stiffness of the intimal flap is accepted as an expression of chronicity^22^, but the classification of intimal flap mobility assessed by IVUS lacks validation in studies so far. The aorta underlies changing flow conditions during the cardiac cycle with constantly pulsatility^23^. We classified non-mobility as ≤3 mm and a real-time intimal flap movement of >3 mm. The cut-off value for mobility visualized by IVUS was defined as <10% of the total aortic diameter, which is more comparable to pulsatility.

For the evaluation of potential short-term benefits of IVUS-assessed intimal flap mobility, the degree of true-lumen expansion was used as an indicator for aortic remodeling. Both groups showed an expansion of true lumen after TEVAR^24^, but the mobile intimal flap group had a more extended absolute and relative expansion of the true lumen, indicating improved aortic remodelling. We performed the evaluation on multiple cross-sectional levels, including the distal stent-graft end, the pulmonary artery and the diaphragm, as previously described^12^. Only the absolute true lumen expansion at the level of pulmonary artery did not differ between both groups. The aortic diameter showed an increased expansion. To account for diameter changes in the descending aorta, the absolute true lumen expansion and the ratio between true lumen and aortic diameter as relative expansion were also included.

TEVAR is a well-established therapeutic modality for treating patients with chronic Type B aortic dissection, but its long-term results are heterogeneous with respect to aortic-related complications or deaths^25^. The overall mortality was low (9.6%) and did not differ significantly between groups. Half of the deaths were due to non-procedural reasons (cancer-related disease and non-procedure-associated stroke), the other half were within 30 days of procedure and were due to major bleeding and septic shock. Our results are in line with previous reports^25^.

The overall re-intervention rate in our study was 21.2%, which was comparable to results from a previous study^26^. Differences in aortic remodeling relating to acute and chronic aortic dissections are believed to be a major factor influencing the uncertainty of clinical outcomes^27^. The fast true lumen expansion and collapse of the false lumen are basically seen in acute aortic dissection, whereas the lack of false lumen thrombosis might promote an adverse clinical long-term outcome in chronic dissections^28^. A previous study has discussed changes in the intimal flap during the process of chronification, resulting in the failure of re-adaptation of the flap towards the aortic wall during TEVAR^29,30^ and under certain circumstances, the need for re-intervention.

The assessment of intimal flap mobility by IVUS in chronic Type B aortic dissection may be helpful in the establishment of personalized treatment strategies to determine the optimal timeframe for TEVAR.

All patients received TEVAR due to clinical indication. The TEVAR procedures were all elective and so did not include emergency cases. Although the IVUS procedure does not take more than 5 minutes and we saw no IVUS-related complications, IVUS increases the procedural time, which should be taken into account. This study is the first to evaluate flap mobility by IVUS, but included a small cohort of 52 patients. Therefore, concluding general recommendations from our results is difficult. Differences in long-term outcome between mobile and non-mobile intimal flap would be surely of interest.

It should be noted that although IVUS may be a useful supplementary tool for flap characterization in selected cases due to the option of dynamic flap assessment, it will not replace CTA. The routine use of IVUS in chronic dissections is likely to be limited due to cost and the invasive nature of the procedure. Additionally, the link between flap mobility and re-intervention indicates the risk of secondary, multifactorial changes including impaired mobility.

Since intimal flap mobility might not be the only marker for the level of chronicity, identifying further promoters of preserved aortic remodelling capacity are needed to be identified.

## Conclusion

High intimal flap mobility in chronic Type B aortic dissection is associated with improved aortic remodelling, seen as extended true lumen expansion, and is related to a better short-term outcome regarding the re-intervention rate after TEVAR. Through the IVUS-assessment of intimal flap mobility in chronic Type B aortic dissection may be helpful in identifying patients with better aortic remodelling after TEVAR.
